# New Technique, New Antibody: Phage Display Identifies Kelch‐Like Protein‐11 Antibodies

**DOI:** 10.1002/mdc3.13136

**Published:** 2020-12-30

**Authors:** Guillermo Delgado‐García, Bettina Balint

**Affiliations:** ^1^ Department of Clinical Neurosciences, Cumming School of Medicine University of Calgary Calgary Alberta Canada; ^2^ Department of Clinical and Movement Neurosciences UCL Queen Square Institute of Neurology London UK; ^3^ Department of Neurology University Hospital Heidelberg Heidelberg Germany

**Keywords:** KLHL11, ataxia, paraneoplastic, autoimmunity, programmable phage display

Mandel‐Brehm C, Dubey D, Kryzer TJ, et al. Kelch‐like Protein 11 Antibodies in Seminoma‐Associated Paraneoplastic Encephalitis. *N Engl J Med* 2019; 381: 47–54.

The spectrum of new autoantibodies keeps expanding. Last year, Mandel‐Brehm and colleagues discovered Kelch‐like protein 11 (KLHL11) as a novel antigen for paraneoplastic cerebellar ataxias using T7 phage display, a relatively new method.^1^ Bacteriophage (phage) display was first described by George Smith, for which he was awarded the 2018 Nobel Prize in Chemistry.^2^ In 2011, a modified human programmable T7 display system engineered to screen for novel antigens was described,^2^ an approach now used by Mandel‐Brehm and colleagues.^1^, ^2^

Since the original description of anti‐KLHL11 disease, based on an index case and 12 additional male patients, 72 additional patients (16 women, 22%) have been reported.^3^, ^4^, ^5^ It appears that, with an estimated prevalence of 1.4 per 100,000 people,^1^ anti‐KLHL11 disease is one of the most common paraneoplastic syndromes. For comparison, the prevalence of Ri autoantibodies is ~0.6 per 100,000 people.^6^

The core phenotype is rhombencephalitis, most frequently presenting with cerebellar signs, often with additional brainstem findings (eg vertigo, tinnitus, hearing loss, diplopia); with subsequently identified cases, there is a widening spectrum including also opsoclonus‐myoclonus, encephalopathy, myeloneuropathy, and cervical amyotrophy.^3^, ^5^

The clinical onset is usually in early‐middle adulthood (range 9–76 years). There is a strong tumor association, mostly with germ cell tumors, especially (extra‐)testicular seminomas. Much more rarely, lung cancer and chronic lymphocytic leukemia are found.^3^, ^5^ MRI findings are diverse, ranging from normal to cerebellar atrophy, cerebellar nuclei T2‐hyperintensities, leptomeningeal enhancement, or mesiotemporal abnormalities. When reported, CSF is abnormal with intrathecal IgG synthesis, hyperproteinorrachia or pleocytosis. Often, there are >8 unmatched oligoclonal bands, suggesting intrathecal antibody production. This may be a diagnostic clue and is in line with the presence of concomitant autoantibodies, seen in 44% of the patients, mainly against Ma2 and NMMDAR, as expected in patients with seminomas and teratomas.^3^

Median serum and CSF titers of KHLH11 autoantibodies are high (1:30,720, range: 1:960–1:245,760) and greater than 1:640, respectively.^5^ KLHL11 autoantibodies target an intracellular protein and, just as the classic paraneoplastic autoantibodies (eg Hu, Yo, Ri), are an epiphenomenon of a T‐cell mediated process. Biopsied, actively inflammatory lesions show a T cell‐predominant inflammation and non‐necrotizing granulomas, while autopsy material with Purkinje neuronal loss and Bergmann gliosis shows subsequent extensive neuronal loss.^5^

Immunotherapy and/or cancer treatment stabilizes or improves the disease course in 58% of patients.^5^ The lack of a detectable testicular cancer seems to have a worse functional prognosis; overall, long‐term outcomes are similar to those of anti‐Ma2 encephalitis.^5^

In conclusion, KLHL11 autoantibodies are the biomarker of a relatively frequent paraneoplastic rhombencephalitis. They should be considered in the workup of acquired ataxias, particularly if there is additional vertigo, hearing loss or tinnitus. Tumor screening is obligatory, as most patients have germ cell (and rarely, other) tumors. Early tumor treatment and immunotherapy are important to try to improve, or at least stabilize, the course of a potentially disabling condition. Lastly, the discovery of KLHL11 autoantibodies illustrates how the spectrum of neuronal autoantibodies keeps expanding, now with the use of a new technology.

## Author Roles

(1) Research Project: A. Conception, B. Organization, C. Execution; (2) Statistical Analysis: A. Design, B. Execution, C. Review and Critique; (3) Manuscript: A. Writing of the First Draft, B. Review and Critique.

G.D.‐G.: 3A

B.B.: 1A, 3B

## Disclosures

### Ethical Compliance Statement

The authors confirm that the approval of an institutional review board and patient consent were not required for this work. We confirm that we have read the Journal's position on issues involved in ethical publication and affirm that this work is consistent with those guidelines.

### Funding Sources and Conflicts of Interest

No specific funding was received for this work. The authors declare that there are no conflicts of interest relevant to this work.

### Financial Disclosures for Previous 12 Months

The authors declare that there are no additional disclosures to report.
